# Duplication of a germline promoter downstream of the *IgH* 3′ regulatory region impairs class switch recombination

**DOI:** 10.1038/s41598-018-27448-4

**Published:** 2018-06-15

**Authors:** Joana M. Santos, Fatima-Zohra Braikia, Chloé Oudinet, Dania Haddad, Caroline Conte, Audrey Dauba, Ahmed Amine Khamlichi

**Affiliations:** 1 Institut de Pharmacologie et de Biologie Structurale, Université de Toulouse, CNRS, Université Paul Sabatier, 31077 Toulouse, France; 2 Present Address: Inserm, UMR-1048, Institut des Maladies Métaboliques et Cardiovasculaires, Toulouse, France; 3 Present Address: FMPU, Dasman Diabetes Institute, Kuwait city, Kuwait

## Abstract

During an adaptive immune response, B cells can change their surface immunoglobulins from IgM to IgG, IgE or IgA through a process called class switch recombination (CSR). Switching is preceded by inducible non-coding germline transcription (GLT) of the selected constant gene(s), which is largely controlled by a super-enhancer called the 3′ regulatory region (3′RR). Despite intense efforts, the precise mechanisms that regulate GLT are still elusive. In order to gain additional insights into these mechanisms, we analyzed GLT and CSR in mutant B cells carrying a duplication of the promoter of the α constant gene (Iα) downstream of 3′RR. Duplication of the Iα promoter affected differently GLT and CSR. While for most isotypes a drop in GLT was accompanied by a decrease in CSR, that was not the case for switching to IgA, which diminished despite unchanged GLT. Unexpectedly, there was no obvious effect on GLT and CSR to IgG3. Remarkably, specific stimuli that normally induce switching to IgG2b had contrasting effects in mutant B cells; Iγ2b was now preferentially responsive to the stimulus that induced Iα promoter. We propose that one mechanism underlying the induced 3′RR-mediated activation of GL promoters involves, at least in part, specific transcription factories.

## Introduction

**Figure 1:**
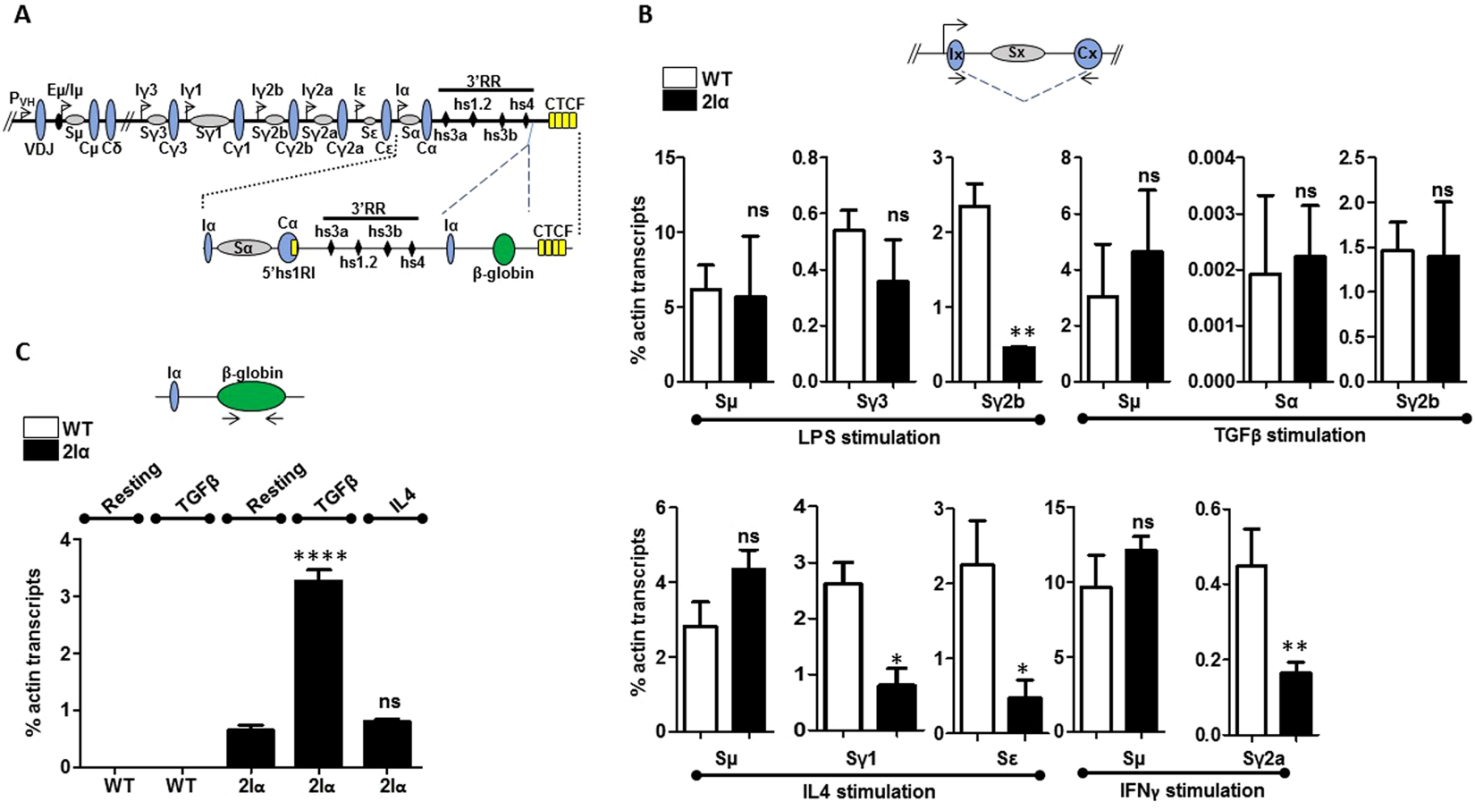
Decreased GLT of specific isotypes in mutant B cells. (**A**) Scheme of the rearranged *IgH* locus in 2Iα mice. The inserted transcription unit contains the mouse Iα GL promoter followed by the terminal intron and exon of the human β-globin gene. The localization of the 5′hs1RI CTCF insulator within the α constant gene is shown as a rectangle (not all CTCF sites downstream of the 3′RR are shown). (**B**) Analysis of GLT in activated B cells. Purified CD43^−^ WT and 2Iα splenic B cells were stimulated for 2 days with the indicated treatments. Total RNA was reverse-transcribed, and the spliced GL transcript levels quantified by qRT-PCR (n = 3). Differences between values from WT and mutant mice were evaluated by a two-tailed t test and error bars represent SD. ns for not significant, *p < 0.05 and **p < 0.01. (**C**) Analysis of β-globin transcript levels. Purified CD43^−^ WT and 2Iα splenic B cells were stimulated, or not (resting), for 2 days with the indicated treatments. Total RNA was reverse-transcribed, and the transcript levels quantified by qRT-PCR (n = 4). ns for not significant, ****p < 0.0001.

Upon antigen challenge, B cells can undergo a recombination process named class switch recombination (CSR). CSR occurs exclusively at the *IgH* locus and leads to a shift in immunoglobulin (Ig) isotype expression from IgM to IgG, IgE or IgA. Recombination involves highly repetitive DNA sequences called switch (S) sequences, located upstream of the constant exons. The donor S region is invariably Sμ and the downstream acceptor S region is chosen depending on the nature of the extracellular stimulus (cytokine, mitogen, antigen…)^1^. The type of signal received by the B cell mobilizes different signaling pathways, ultimately resulting in the recruitment of a specific set of transcription factors that can suppress or induce transcription from constant genes promoters (*e.g*.^2,3^). These so-called germline (GL) or I promoters are localized upstream of all constant genes except Cδ (see Fig. 1A). Non-coding, GL transcription (GLT) initiated from the selected promoter(s) runs across the corresponding S region and generates secondary structures that provide the substrate for AID (Activation-induced cytidine deaminase), which initiates DNA breaks, ultimately leading to a fusion between Sµ and the partner S sequence^1^.

Various mutational studies have shown that GLT is regulated by the inducible 3′ regulatory region (3′RR), which contains four enhancers (hs3a, hs1–2, hs3b, and hs4) located downstream of the *IgH* locus^4^. The 3′RR has been shown to effect a long-range enhancing activity on the multiple I promoters as well as on ectopic promoters when inserted upstream of the 3′RR, probably involving a competition between target promoters for 3′RR activity (*e.g*.^5–8^). In this context, replacement of Iγ3 by Iγ1 promoter, resulting in a duplication of Iγ1 promoter upstream of the 3′RR, led to a decrease in GLT from the upstream, ectopic Iγ1, but not from the downstream, endogenous, Iγ1 promoter^7^. This raises the question as to how duplication of a GL promoter downstream of the 3′RR would affect activation of the upstream promoters.

We have recently generated a mouse line (called 2Iα line) in which a chimeric transcription unit driven by the Iα promoter has been inserted downstream of the 3′RR so that the 3′RR is now flanked by identical Iα promoters^9^ (Fig. 1A). In this study, we analyzed the effect of the mutation on GLT and CSR. We report the striking finding that the insertion of the Iα promoter downstream of the 3′RR reduces GLT and CSR to most, but not all, isotypes, and that the stimuli that normally induce the 3′RR-mediated activation of Iγ2b promoter now have contrasted effects.

## Results and Discussion

### Duplication of the Iα promoter differentially affects GLT

In order to determine if insertion of the Iα promoter downstream of the 3′RR (Fig. 1A) had any effect on GLT, we quantified the levels of the pre-switch transcripts that initiate from the different I promoters and terminate downstream of the corresponding constant regions (Fig. 1B, top scheme). With the exception of the constitutive Sµ GL transcripts, derived from the Eµ/Iµ promoter, GLT across all other S regions is induced upon appropriate stimulation of splenic B cells. Therefore, sorted B cells from WT and 2Iα littermates were stimulated with LPS (to induce GLT of Sγ3 and Sγ2b), with IL4 (to induce GLT of Sγ1 and Sε), with TGFβ (to induce GLT of Sγ2b and Sα), or with IFNγ (to induce GLT of Sγ2a). At day 2 post-stimulation, total RNAs were extracted, reverse-transcribed and analyzed by qPCR. We found no difference in Sµ transcript levels regardless of stimulation conditions (Fig. 1B). In contrast, we observed differential effects on downstream GL transcripts.

Strikingly, upon LPS stimulation, while Sγ3 transcript levels were comparable between 2Iα and WT B cells, there was a significant decrease in the levels of Sγ2b transcripts in the mutant cells (Fig. 1B). Upon IL4 stimulation, we found reduced levels of Sγ1 and Sε transcripts in 2Iα cells (Fig. 1B). Transcript levels of Sγ2a, as induced by IFNγ, were also diminished in 2Iα cells. Surprisingly, we did not detect any significant decrease in the levels of Sγ2b transcripts, when the 2Iα B cells were stimulated with TGFβ. The levels of Sα were also similar between WT and 2Iα B cells, as previously reported^9^. Although already active in 2Iα resting B cells, the ectopic promoter was further induced by TGFβ stimulation, as measured by β-globin transcript levels. This change was not significant with the other stimulation conditions (Fig. 1C and data not shown), suggesting that the Iα promoter retained its specific TGFβ-responsiveness at the ectopic site.

Thus, insertion of the Iα promoter downstream of the 3′RR resulted in differential effects on pre-switch GLT. While Sγ1, Sε, and Sγ2a transcript levels were reduced, Sγ3 and Sα transcript levels were unaffected. The effect on Sγ2b transcription depended on the stimulation condition; Sγ2b transcript levels were decreased upon LPS stimulation but were unaffected upon TGFβ stimulation.

### Insertion of Iα promoter downstream of the 3′RR affects CSR

**Figure 2:**
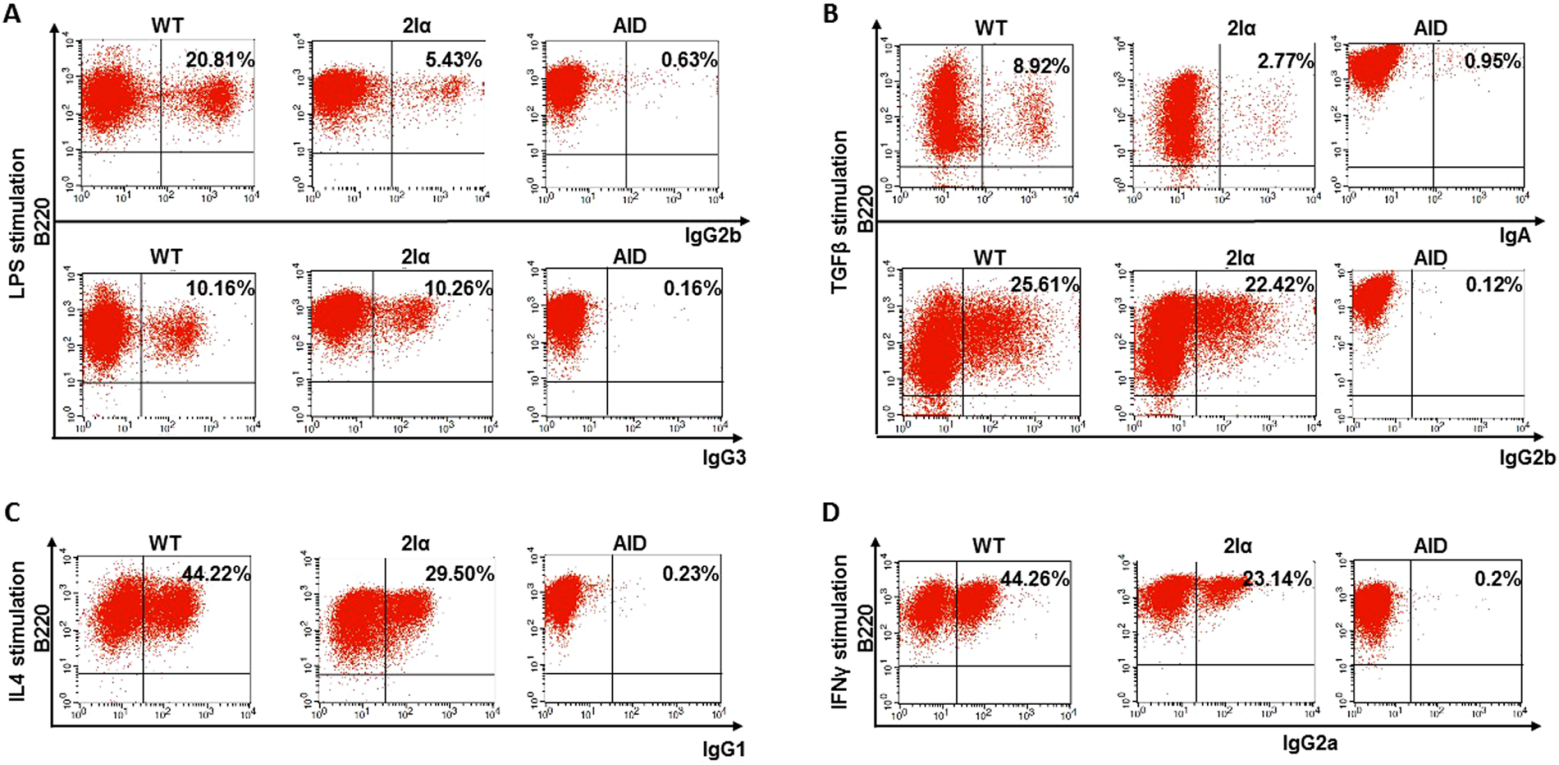
Decreased CSR to most isotypes in mutant B cells. CD43^−^ sorted splenic B cells of WT or 2Iα mice were induced to switch in the presence of LPS (**A**), TGFβ (**B**), IL4 (**C**) and IFNγ (**D**) and at day 4 post-stimulation, the cells were stained with the indicated antibodies and analyzed by FACS.

Because pre-switch GLT is a pre-requisite for CSR, we asked whether duplication of the Iα promoter would affect CSR similarly to GLT. CSR was analyzed by monitoring surface expression of Ig isotypes by FACS. Throughout, AID-deficient B cells were used as negative controls as they are unable to initiate CSR (Fig. 2A). Following LPS stimulation, while CSR to IgG3 was unaffected, CSR to IgG2b was clearly decreased (Fig. 2A). CSR to IgG1 (Fig. 2B) and to IgG2a (Fig. 2C) was also reduced following IL4 and IFNγ stimulation, respectively. Strikingly, while CSR to IgG2b was unaffected upon TGFβ stimulation, CSR to IgA was clearly diminished (Fig. 2D).

**Figure 3:**
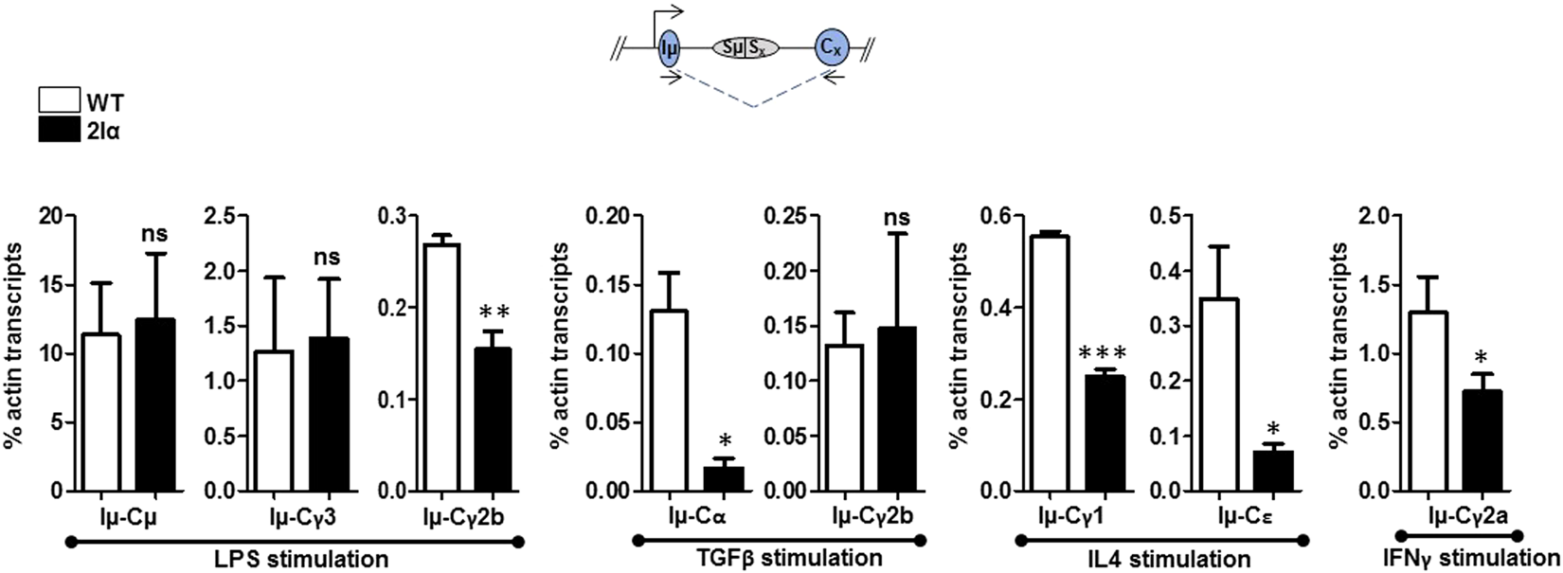
CD43^−^ sorted splenic B cells of WT or mutant mice were induced to switch to the different isotypes and at day 4 post-stimulation, RNA was collected and reverse-transcribed. The levels of Iµ-Cx post-switch transcripts, as determined by qRT-PCR are indicated (n = 3). Differences between values from WT and mutant mice were evaluated by a two-tailed t- test and error bars represent SD. ns for not significant, *p < 0.05, **p < 0.01, and ***p < 0.001.

The impact of the duplication of Iα promoter on CSR was also analyzed by quantifying the levels of post-switch transcripts that initiate from the Eµ/Iµ promoter and terminate downstream of the switched constant region (Fig. 3C, top scheme). The levels of post-switch transcripts were quantified by qRT-PCR. For this purpose, total RNAs were collected, reverse-transcribed and analyzed by qPCR at day 4 post-stimulation. As shown in Fig. 3, the data obtained for post-switch transcription mirrored perfectly that obtained by FACS. Upon LPS stimulation, Iµ-Cγ3 transcript levels did not vary between mutant B cells and WT controls. Iµ-Cγ2b transcript levels, in contrast, were reduced in 2Iα B cells (Fig. 3A). Iµ-Cγ1 and Iµ-Cε transcript levels were both reduced in IL4-activated mutant B cells (Fig. 3B). Similarly, there was a drop in Iµ-Cγ2a transcript levels following IFNγ treatment (Fig. 3A). Upon TGFβ stimulation, while Iµ-Cγ2b transcript levels did not significantly vary, Iµ-Cα levels were clearly decreased (Fig. 3A).

Altogether, the above data shows that insertion of Iα promoter downstream of the 3′RR reduces CSR to IgG1, IgE, IgG2a, and IgA. In contrast, CSR to IgG3 is not affected. CSR to IgG2b is reduced in LPS-activated mutant B cells but is unaffected upon TGFβ stimulation.

Thus, insertion of Iα GL promoter downstream of the *IgH* 3′RR differentially impairs CSR. The effect seen on CSR to most isotypes can be explained by the impact of the insertion on GLT; reduction of CSR to IgG1, IgG2a, and IgE is most likely due to reduced pre-switch transcription of Sγ1, Sγ2a, and Sε respectively. On the other hand, CSR to IgG3 was unaffected by Iα promoter duplication, and this correlated well with normal levels of Sγ3 GL transcripts in mutant cells. This remarkable finding indicates that premature activity of the ectopic Iα promoter^9^ did not prevent 3′RR-mediated activation of Iγ3 upon LPS stimulation. This was somewhat unforeseen because previous studies have shown that partial or complete deletion of the 3′RR deeply affects switching to IgG3^6,8^. One possible explanation is that the Iγ3 promoter and 3′RR are already in close proximity in resting B cells^9^, and, possibly due to competition between promoters for 3′RR activity^5^, activation of the Iγ3 promoter is initially favored in detriment of Iγ2b.

In contrast, CSR to IgA and IgG2b displayed unexpected features. CSR to IgA was diminished despite normal levels of pre-switch Sα GLT. The effect of the mutation on CSR to IgG2b depended on the nature of the inducer. Upon LPS stimulation, CSR to IgG2b was reduced, which correlated with reduced Sγ2b GL transcript levels. In contrast, following TGFβ stimulation, CSR to IgG2b was unimpaired, which correlated with normal Sγ2b GL transcript levels. Thus, with regard to the association between GLT and CSR, the only discrepancy concerns CSR to IgA. One possibility could be that the highly transcribed ectopic unit actively recruits AID, which leads to deletion of Cα following switch-like events downstream of the 3′RR, reminiscent of locus suicide recombination^10^. This is unlikely because the ectopic unit does not contain any switch sequence or repeat motif that would provide an optimal substrate for AID to initiate DNA breaks. More importantly, the normal levels of CSR to IgG2b in TGFβ-activated B cells argue against such scenario. In this context, reduced CSR to IgA, in the presence of normal levels of Sα transcripts, was also found in mouse B cells deficient for the p50 subunit of NF-κB^11^ or the histone methyl-transferase Suv39h1^12^, and to some extent in 3′RR-deleted and Cohesin-deficient clones of CH12F3 B cell line^13,14^, as well as in some IgA-deficient patients^15^. These observations suggest that, at least for a structurally intact α constant gene, normal levels of GLT are not sufficient for efficient CSR to IgA. Whether this is due to specific features of Sα chromatin is presently unclear.

Interestingly, insertion of the neomycin resistance gene under the control of the phosphoglycerate kinase promoter (PGK-*Neo*^*R*^), downstream of the 3′RR (hs4-NI mice) had no effect on CSR^16^. In this regard, the reduction seen in this study for Sγ2b (upon LPS stimulation), Sγ1, Sγ2a and Sε transcript levels was unexpected. It remains possible that altered local chromatin structure at the different insertion sites of PGK-*Neo*^*R*^ (hs4-NI) and Iα promoter (2Iα) prevents access and interaction of the 3′RR with the upstream I promoters, although this fails to explain why only some promoters are affected. It is also possible that the ectopic insertion had perturbed the 3D structure of the 3′RR *per se*. However, the normal CSR to all isotypes seen in hs4-NI mice and to IgG3 in 2Iα mice suggests otherwise. One plausible explanation is that the induced PGK promoter and the ectopic Iα promoter affect the 3D architecture of the *IgH* locus through different mechanisms. Bioinformatics analysis using JASPAR software revealed the presence of a putative CTCF binding site (TCCACCGGTAGGCGCCA) in the PGK promoter but not in Iα promoter. In this context, CTCF bound to the PGK promoter may interact with CTCF bound to cognate sites downstream of the *IgH* locus^17^ leaving intact the interactions established between the 3′RR and upstream GL promoters. The potential binding of CTCF to the PGK promoter could also explain why insertion of PGK-*Neo*^*R*^ in the *IgH* locus affects transcription from upstream (relative to the insertion site), but not from downstream promoters (discussed in^4,5^).

In the case of 2Iα mice, reduced Sγ2b (in LPS stimulation), Sγ1, Sε and Sγ2a transcript levels may result from the continuous activity of the ectopic Iα promoter under LPS, IL4 and INFγ stimulations. While we cannot formally exclude this possibility, this does not fully explain the observed phenotype because the highest activity of the ectopic Iα promoter (as measured by the human β-globin transcript levels) was detected upon TGFβ stimulation (Fig. 1C), which did not affect Sγ2b and Sα pre-switch GL transcription. Additionally, this cannot explain why Sγ3 GLT was unaffected upon LPS stimulation, whereas Sγ2b GLT was.

An alternative mechanism may be proposed based on the striking finding that Sγ2b transcript levels were reduced in LPS stimulation but were normal in TGFβ stimulation. The latter mobilizes members of the SMAD transcription factors family and other factors such as RUNX3 that bind to their cognate sites at Iγ2b and Iα promoters^18,19^. This raises the possibility that 3′RR-mediated activation of GL promoters occurs in distinct transcription factories, in a stimulus dependent manner. Such “specialized” transcription factories would ensure that GL promoters that share a specific set of transcription factors would preferentially co-localize in the same transcription factory, while GL promoters that do not would be excluded. Correlation between shared transcription factors and shared transcription factories has been established for some regulatory factors such as KLF-1, which plays an important role in the expression of globin genes as well as other genes^20^ and NF-κB for several TNFα-induced genes^21^. Significantly, promoter identity appears to play an important role in this process^22^. In the specific case of 2Iα mice, one speculative scenario is that continuous activity of the ectopic Iα promoter^9^ retains the 3′RR in a transcription factory enriched in TGFβ-induced transcription factors. Promoters that do not respond to TGFβ pathway would be down-regulated. Iγ3 promoter may somehow be an exception because of the 3D proximity with the 3′RR (discussed in^9^). Whether the transcription factors induced by TGF-β also mediate the long-range interactions between the 3′RR and target promoters is presently unknown.

In conclusion, we provided evidence that duplication of the Iα promoter downstream of the 3′RR led to pleiotropic effects on GLT and CSR. For most isotypes, we found a good correlation between decreased GLT and impaired CSR. However, there were remarkable exceptions. GLT that initiates from Iγ3 promoter was normal as was CSR to IgG3. In contrast, CSR to IgA was reduced despite normal levels of pre-switch transcription. Remarkably, Iγ2b promoter which is induced by either LPS or TGFβ stimulation, was activated upon TGFβ stimulation only. Thus, with the exception of Iγ3, GLT derived from all other promoters not induced by TGFβ stimulation was decreased. This indicates that the 3′RR activity is not optimally available to these promoters even in stimulation conditions that normally induce them. Moreover, the case of Iγ2b promoter reveals that repression of these promoters is not irreversible provided they respond to TGFβ stimulation. It thus appears that the active Iα promoter has “sequestered” the 3′RR in a compartment that is permissive to only those GL promoters that respond to the same stimulus as Iα, *i.e*. TGFβ stimulation. Thus, our study suggests that one mechanism underlying the 3′RR-mediated activation of GL promoters involves, at least in part, specific transcription factories. Whether it is the 3′RR that directs the responsive GL promoters to the appropriate transcription factories or the other way around is presently unknown, though our data point to an active role of the GL promoter.

## Materials and Methods

### Mice and ethical guidelines

The WT and mutant mice are of 129 Sv background. All analyses were performed on homozygous 2Iα mice. Three mice of each genotype were used per experiment, and each experiment was repeated at least 3 times. 5–7 weeks old mice were used. All experiments on mice have been carried out according to the CNRS ethical guidelines and were approved by the Regional Ethical Committee (Accreditation N° E31555005).

### Cell sorting and splenic B-cell activation

Splenic single cell suspensions were obtained by standard techniques and B cells were negatively sorted by using CD43-magnetic microbeads and LS columns (Miltenyi) and cultured for 2 days (for pre-switch GLT) and 4 days (for CSR and post-switch GLT), at a density of 5 × 10^5^ cells/ml in the presence of 25 µg/ml of LPS (Sigma) and 3 ng/ml anti-IgD-dextran (Fina Biosolutions) (LPS stimulation); 25 µg/ml LPS and 3 ng/ml anti-IgD-dextran and 20 ng/ml IFNγ (R&D) (IFNγ stimulation); 25 µg/ml LPS and 3 ng/ml anti-IgD-dextran and 25 ng/ml IL4 (eBiosciences) (IL4 stimulation); and 25 µg/ml LPS and 3 ng/ml anti-IgD-dextran and 10 ng/ml IL4 and 5 ng/ml IL5 (R&D) and 5 ng/ml BLys (R&D) and 2 ng/ml TGFβ (R&D) (TGFβ stimulation).

### Flow cytometry

At day 4 post-stimulation, B cells were washed and stained with anti-B220 APC (BioLegend) and either anti-IgG3-FITC (BD-Pharmingen), anti-IgG2b-PE (BioLegend), anti-IgG2a-PE (BioLegend) or anti-IgG1-FITC (BioLegend). Activated B cells from AID-deficient mice were included as negative controls. Data were obtained on 3 × 10^4^ viable cells by using a Coulter XL apparatus (Beckman Coulter).

### qRT-PCR

Total RNAs were prepared from resting splenic B cells, and B cells at day 2 or 4 post-stimulation. Total RNAs were reverse-transcribed (Invitrogen) and subjected to qPCR using Sso fast Eva Green (BioRad). *Actin* transcripts were used for normalization and the results are shown as percentage of actin. (−RT) controls were included in all of the experiments. The primers used have been described^9^.

### Statistical analysis

Results are expressed as mean ± SD (GraphPad Prism), and overall differences between values from WT and mutant mice were evaluated by a two-tailed t test. The difference between means is significant if *p* < 0.05 (*), very significant if *p* < 0.01 (**), and extremely significant if *p* < 0.01 (***).
